# Obstacles to Post-mortem Cornea Donation: A Study From a Tribal Region in Eastern India

**DOI:** 10.7759/cureus.25176

**Published:** 2022-05-20

**Authors:** Suchitra Panigrahi, Bidisha Mahapatra, Sandhyarani Pati, Shibanee Jena, Sunil K Murmu, Punyanshu Mohanty

**Affiliations:** 1 Ophthalmology, Pandit Raghunath Murmu Medical College, Baripada, IND; 2 General Medicine, Maharaja Krishna Chandra Gajapati (MKCG) Medical College and Hospital, Berhampur, IND; 3 Anatomy, Sri Jagannath Medical College and Hospital, Puri, IND; 4 Forensic Medicine and Toxicology, Pandit Raghunath Murmu Medical College, Baripada, IND

**Keywords:** corneal procurement rate, eye benefactor, cornea transplantation, eye donation, post mortem corneal retrieval

## Abstract

Purpose: Studying the cognizance and hindrances of eye benefactors amongst relatives of post-mortem cases in an Indian tertiary referral centre.

Methods: This prospective study was executed at a tertiary hospital. In this examination, the relatives had been approached and counselled systematically. The responses had been noted in a predesigned proforma. Data regarding demographic details, socioeconomic status, prior knowledge of eye benefactor, willingness and reasons for refusing eye benefactor, literacy level, relationship with the deceased patient, and so on were collected.

Results: One hundred fifty-six potential donors had been identified from 845 post-mortem cases. Among these potential donors were 63 women and 93 men. Thirty-eight next of kin had been seen as already cognizant regarding eye benefactor; however, other 118 families were unaware. A total of 109 families refused to donate eyes while other 47 showed willingness for the procedure. It was seen that there was no influence on literacy status, socioeconomic status and prior knowledge regarding the concept of willingness to donate.

Conclusion: Counselling for eye benefactor exercises a crucial role in procuring corneas. Socioeconomic status, literacy and prior understanding of eye donation had no link with donor corneal tissue procurement in our study. Even in families with no prior knowledge and poor socioeconomic status, active counselling can be successful.

## Introduction

Visual impairment is a major health concern worldwide, which can be managed by good quality donor corneal tissue in corneal transplantation. According to the World Health Organisation (WHO) [1], there exist 45 million blind people globally and 5% of which are with corneal diseases [2,3]. The Eye Bank Association of India has made significant efforts over the years to increase the corneal procurement rate, which is currently 49,000 per year [4]. However, because 30,000 cases are added each year, and the quality of procured corneas is not always good, it is estimated that 277,000 donor tissues are required each year to fill the gap between demand and supply of corneal tissue [5,6]. Because the eye benefactor is entirely voluntary, it is critical to educate the general public about their social obligation to the corneal blind. Many eye banks have implemented the Hospital Cornea Retrieval Programme (HCRP), in which Eye Donation Counselors (EDCs) meet families and aggressively educate them about eye benefactor. The knowledge and beliefs of the family, on the other hand, have a substantial impact on consent. Family members' attitudes and knowledge about cornea donation play a significant impact in obtaining favourable authorisation for eye benefactor. However, the attitude of family members is diverse after the loss of a loved one, and making a gift request at that time is the most difficult portion. There have been few studies on the procurement of donor corneas, and there has been hardly any research done on the people of South Asia. This study tries to fill a gap in the literature by looking at the causes and barriers to permission for eye benefactors in patients undergoing post-mortem investigation at a tertiary care hospital in a tribal area of Eastern India.

## Materials and methods

The study included cases brought for post-mortem to the department of forensic medicine at our hospital's mortuary within a year. For this study, tribal people were considered as a sample due to their low socioeconomic status, low literacy rates and also lack of knowledge regarding corneal donation. This helps in understanding whether these three factors affect their choices of corneal donation. The exclusive criteria were >12 hours since death, septicemia, mutilated face, homicide, unclaimed body and the unknown cause of death. The EDCs had previously completed a training programme and were familiar with the operations of the Eye Bank, the HCRP programme, and how to contact the family members of the deceased to counsel the eye benefactor.

The study was carried out in accordance with the principles of the Helsinki Declaration, and approval from the Pandit Raghunath Murmu Medical College Ethics Committee (1520/IEC/PRMMCH/2021) was obtained. The eye bank retrieval team evaluated the patients to determine their appropriateness for eye benefactor. The gender and age of the deceased potential donor, as well as the cause and time of death, were all recorded. Pathological death, which comprised cases such as cardiac arrest, heart attack, cerebrovascular accident, malignancy and multiple organ failure, was divided into two categories. The second category was an accidental death, which included deaths caused by drowning, hanging, murder, falls from great heights, fire and poisoning. EDCs approached the deceased's family relatives to discuss the eye benefactor. These people were identified as potential eye donors.

Before requesting an eye benefactor, information on the family, the deceased's relationship and the deceased's level of acceptance of death were acquired. The cause of death, literacy level, family per capita income, cognizance of eye benefactor, willingness for eye benefactor and grounds for denial of eye benefactor were all recorded. Families who refused to donate their eyes were thanked, and important information regarding eye benefactors was left with them in case they changed their minds. Families who were unsure about donating were given enough time to discuss their concerns with EDCs or other family members. When the family agreed to donate an eye, written approval was obtained. Donation consent was always obtained from the lawful next of kin.

Whole-globe enucleation was conducted under aseptic conditions, and the enucleated eyes were transported in a moist chamber to the closest eye bank for storage in the McCarey-Kaufman medium. The Modified Kuppusamy Index score was used to assess socioeconomic status [7]. Literacy was defined as any participant above the age of five who could read and write in the native language completely.

Statistical analysis

Statistical Package for the Social Sciences (SPSS) statistical software version 22.0 (IBM Corp., Armonk, NY) was used for statistical analysis. For continuous data, mean and standard deviation were recorded, while for categorical variables, percentages were reported. The chi-square test was used to compare categorical data between the two groups, while the independent-sample t-test was used to analyse continuous variables. The level of statistical significance was set at 5% (p < 0.05).

## Results

A total of 156 people were examined as potential donors amongst 845 autopsies performed in the specified institution between January 2021 and December 2021. Table 1 shows that the average age of potential donors' next of kin was 39 ± 11.03 years (range 19-80 years), with 93 men and 63 women. Age and gender had no significant relationship with approval for eye benefactor (p = 0.52, p = 0.15, respectively).

**Table 1:** Consolidated profile of the potential donors

	Donor	Non-donor	p-Value
Age	43.34 ± 13.66	41.22 ± 14.54	0.39
Gender			
Male	34 (72.3%)	73 (66.9%)	0.50
Female	13 (27.6%)	36 (33%)	
Cause of death			
Pathological	6 (12.7%)	15 (13.7%)	0.86
Accidental	41 (87.2%)	94 (86.2%)	

**Figure 1:**
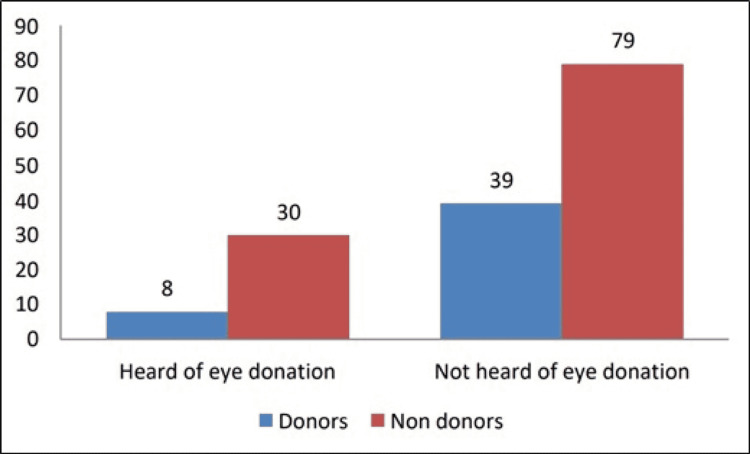
Awareness of eye benefactor and consent of participants

The participant's relationship with the deceased who was primarily responsible for the decision of donating an eye was spouse (23%), child (19.8%), sibling (14.7%) and parent (19.8%). The relationship of the participant with the deceased did not showcase any significant correlation with the idea of donating eyes (p = 0.79). Thirty-eight (24.3%) of the participants were already aware of the eye benefactor, while 118 (75.6%) did not. Figure 1 illustrates that on a scale of 0-90, eight out of the 38 (21%) participants who knew about the eye benefactor agreed to donate, while 39 out of 118 (33%) with no information agreed to donate.

One hundred and fifteen (73.7%) households had a lower socioeconomic position, whereas 41 (26.2%) families had a higher socioeconomic status. There were 127 (81.4%) literate participants and 29 (18.5%) illiterate persons. As seen in Table 2, there was no significant distinction in the approval rate for eye benefactors based on the participants' literacy (p = 0.31) or socioeconomic category (p = 0.79).

**Table 2:** Consolidated profile of participants

Profile	Consent	Non-consent	p-Value
Age	40.62 ± 10.82	39.38 ± 11.14	0.52
Gender			0.15
Male	24 (51%)	69 (63.3%)	
Female	23 (48.9%)	40 (36.6%)	
Relationship with the deceased			0.79
Spouse	16 (34%)	20 (18.3%)	
Parent	10 (21.2%)	21 (19.2%)	
Children	12 (25.5%)	19 (17.4%)	
Siblings	9 (19.1%)	14 (12.8%)	
Socioeconomic status			0.79
Low	34 (72.3%)	81 (74.3%)	
High	13 (27.6%)	28 (25.6%)	
Literacy rate			0.31
Literate	36 (76.5%)	91 (83.4%)	
Illiterate	11 (23.4%)	18 (16.5%)	
Religion			0.82
Hinduism	46 (97.8%)	106 (97.2%)	
Islam	1 (2.1%)	3 (2.7%)	

**Figure 2:**
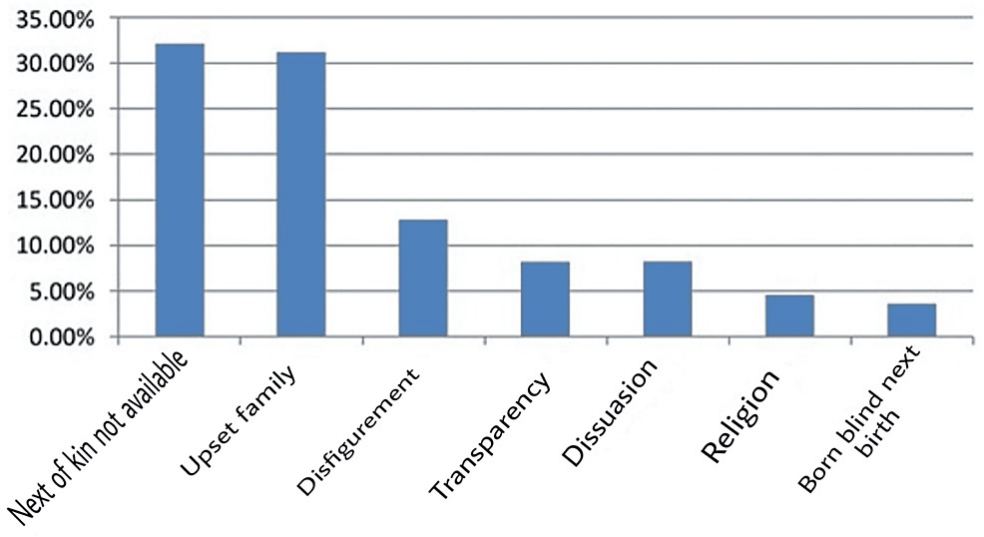
Barriers in decision making for eye benefactor

Out of the 109 cases where a cornea could not be obtained, 35 cases were because of the absence of legal next of kin at the site of the post-mortem, 34 cases refused to discuss eye benefactor amidst the stressful situation of death, 14 (12.8%) were concerned regarding the facial disfigurement following enucleation, 9 (8.2%) refused because of non-transparency in tissue utilisation and fear of organ trafficking, and 9 (8.25%) because of dissuasion by another family. Figure 2 reveals that on a scale of 0.00-35.00%, 5 (4.5%) were because of religious beliefs and 4 (3.6%) because of the idea that if eyes are donated at the moment of death, one will be born visually impaired in the next birth.

## Discussion

Globally, a scarcity of cornea is a serious impediment to restoring eyesight in the blindness of the cornea. Much work remains to be done to raise awareness and dispel myths about eye benefactors. Previous research has shown the effect of EDCs in positively influencing families' decisions to donate their eyes.

In our study, 30.1% of counselling families agreed to donate their eyes, which is comparable to prior studies [8-11]. Because of greater literacy rates and family socioeconomic levels, the consent rate in industrialised nations is slightly higher [12,13]. In our study, socioeconomic status and family literacy had no effect on the desire to use eye donation (p = 0.31 and p = 0.79, respectively). This is consistent with the findings of Tandon et al. [8] and Acharya et al. [14].

Because our study was done in a location with a largely tribal population, our awareness rate was 24.3%, which was lower than in many other studies [11,12,15,16]. Acharya et al. [14] stated that healthcare staff, friends and family, television and mass media were all important sources of raising awareness. As a result, these steps should be pushed in underserved areas of the country as well, including the celebration of eye benefactor awareness week and the organisation of awareness-raising camps. According to Kumar and Ravendran [17], educational status had a beneficial impact on the apprehension of the eye benefactor but had no statistically significant effect on willingness to donate eyes.

In our study, the mean age of the deceased was 41.86 ± 14.27 years, and age did not significantly affect the willingness of participants to eye benefactor (p = 0.39), similar to the study by Acharya et al. [14], but several other studies [9,15,18] have found a significant relationship between age and decision for eye benefactor, with the elderly being more willing than the young. In the same vein as Acharya et al. [14]. we too found no significant relationship between the deceased's cause of death and the participant's willingness to donate an eye (p = 0.86). In our study, 59.6% of participants were male and 40.3% were female, and gender had no significant relationship with the choice to donate an eye (p = 0.15). In contrast to a previous study [14], individuals accompanying the deceased were largely male since, in most regions of India, societal beliefs dictate that males accompany the corpse to the post-mortem location.

Research like Acharya et al.'s [14] and Bhandary et al.'s [19] have found that parents are more ready to donate the deceased's eyes than children, spouses or siblings. In our study, however, parents, children, spouses and siblings were virtually equally ready to donate the eyes of the deceased (p = 0.79).

The biggest barriers to donor eye procurement are logistical issues and a failure to seek donation [20]. Several factors influence people's willingness to donate organs or tissues. In our analysis, the most common cause for corneal non-procurement was the absence of legal next of kin at the post-mortem site (32.1%). The most common reason for denial was families' refusal to discuss eye benefactor (31.1%) owing to emotional stress. Tandon et al. [8] also observed that the main reason for refusal was the participants' refusal to discuss eye benefactor (42.5%). Various reasons included the perception that enucleation would disfigure the face, transparency in tissue utilisation and concern of organ trafficking, dissuasion by relatives, and other religious and cultural views. Lawlor et al. [21] and Golchet et al. [22] both expressed concern about facial damage following donation. It was discovered that certain communities, such as Muslims, had strong beliefs that served as a barrier to eye benefactor [23,24]. Tandon et al. [8] also found that 5% of families were concerned regarding organ trafficking. In tribal areas like ours, the active promotion of eye benefactor through audio-visual aids such as short video clips and the display of other instructional information at the location where relatives wait to receive the body after autopsy may assist overcome this barrier.

Despite the findings of this study, there were certain limitations too. The variability of the recipient's eyes, including diverse corneal diseases of the anterior corneal stroma, is a restriction. The inclusion of a larger, more homogeneous group of eyes appears to be difficult.

## Conclusions

Our research emphasises the impact of EDCs in aiding the eye benefactor movement. This study from the country's tribal areas demonstrates that literacy, social position and prior knowledge of eye benefactors have no association with willingness to donate eyes. Proactive counselling by an eye benefactor counsellor can help to overcome emotional, cultural and religious beliefs, which can considerably improve the procurement of donor corneal tissue. The impediments to eye benefactor are not simply cultural or religious in nature, but also stem from misunderstandings about the use of donated tissue. There is a need to clarify these misconceptions, which can be accomplished by holding eye benefactor awareness camps and disseminating information about it through mass media and communication tools, particularly in healthcare institutions.
